# Alternative Splicing Regulation of Anthocyanin Biosynthesis in *Camellia sinensis* var. *assamica* Unveiled by PacBio Iso-Seq

**DOI:** 10.1534/g3.120.401451

**Published:** 2020-06-09

**Authors:** Lijiao Chen, Xingyun Shi, Bo Nian, Shuangmei Duan, Bin Jiang, Xinghua Wang, Caiyou Lv, Guanghui Zhang, Yan Ma, Ming Zhao

**Affiliations:** * College of Longrun Pu-erh Tea, State Key Laboratory of Conservation and Utilization of Bio-resources in Yunnan, and National & Local Joint Engineering Research Center on Gemplasm Innovation & Utilization of Chinese Medicinal Materials in Southwest China, Yunnan Agricultural University, Kunming, Yunnan 650201, China,; ^†^ Guizhou Tea ResearchInstitute, Meitan, Guizhou 564100, China,; ^‡^ Tea Technique Extension Station of the Agriculture Bureau of Dehong, Yunnan, 678400, China, and; ^§^ Tea Seed Multiplication Farm of Pu'er Yunnan, Pu'er 665000, China

**Keywords:** anthocyanins, PacBio Iso-Seq, alternative splicing events

## Abstract

Although the pathway and transcription factor regulation of anthocyanin biosynthesis in tea plants [*Camellia sinensis* (L.) O. Ktze] are known, post-transcriptional regulation mechanisms involved in anthocyanin accumulation have not been comprehensively studied. We obtained the full-length transcriptome of a purple cultivar (‘Zijuan’) and a normal green cultivar (‘Yunkang 10#) of *C. sinensis* var. *asssamica* (Masters) showing different accumulation of anthocyanins and catechins through PacBio isoform sequencing (Iso-Seq). In total, 577,557 mapped full-length cDNAs were obtained, and 2,600 average-length gene isoforms were identified in both cultivars. After gene annotations and pathway predictions, we found that 98 key genes in anthocyanin biosynthesis pathways could have undergone alternative splicing (AS) events, and identified a total of 238 isoforms involved in anthocyanin biosynthesis. We verified expression of the *C4H*, *CHS*, *FLS*, *CCOM*, *F3′5’H*, *LAR*, *PAL*, *CCR*, *CYP73A13*, *UDP75L12*, *UDP78A15/UFGT*, *UDP94P1*, *GL3*, *MYB113*, *ANR*, *ANS*, *F3H*, *4CL1*, *CYP98A3/C3H*, *CHI*, *DFR* genes and their AS transcripts using qRT-PCR. Correlation analysis of anthocyanin biosynthesis and gene expression results revealed that *C4H1*, *FLS1*, *PAL2*, *CCR2*, *UDP75L122* and *MYB113-1* are crucial AS transcripts for regulating anthocyanin biosynthesis in *C. sinensis var. assamica*. Our results reveal post-transcriptional regulation of anthocyanin biosynthesis in tea plants, and provide more new insights into the regulation of secondary metabolism.

Anthocyanin, an important type of flavonoid, produces a wide range of colors from orange/red to violet/blue in flowers, leaves and fruit (Tanaka *et al.* 2008; Giovana *et al.* 2019). Additionally, anthocyanins may contribute to the observed human health benefits of consuming red or purple commercial crops, which can be used as dietary nutraceutics with anti-aging and anti-cancer effects (Winkel-Shirley 2001). The tender shoots of the tea plant are usually yellow and green, with 12–24% of dry matter being catechins and 0.01% being anthocyanins (Wang and Ho 2009). In purple-leaf genotypes of the ‘Zijuan’ (ZJ) cultivar of *C. sinensis* var. *asssamica* (Masters) Kitamura, the anthocyanin level is highest in immature shoots (0.58–6.16%) (Li *et al.* 2016). In recent years, anthocyanin-rich tea products have become increasingly popular owing to their attractive colors and suggested human health benefits, which mainly manifest through antioxidant properties, reducing chronic and degenerative diseases (Achterfeldt *et al.* 2015; Charepalli *et al.* 2015).

The anthocyanin biosynthetic pathway is clear in plants, and three early biosynthetic genes, *chalcone synthase* (*CHS*), *chalcone isomerase* (*CHI*) and *flavanone 3-hydroxylase* (*F3H*), and four late biosynthesis genes, *flavonoid 3′-hydroxylase* (*F3′H*), *dihydroflavonol reductase* (*DFR*), *anthocyanidin synthase* (*ANS*) and *flavonoid 3-O-glucosyltransferase (UFGT)*, have been identified as structural genes for regulating anthocyanin accumulation in many tea plants, including ‘Mooma 1’ (He *et al.* 2018), ‘Shuchazao’, ‘Fudingdabaicha’, ‘Longjing43’, ‘Xinyangdaye’, ‘Zhongcha302’, ‘Huangkui’, ‘Ziya’, ‘TEENALI’, ‘Hongye2’ (Wang *et al.* 2018a) and ‘Wuyiqizhong C18’ (Zhou *et al.* 2015). Additionally, transcription factors (TFs) including those of the MYB (R2R3-MYB and R3-MYB TFs), bHLH and WD40 families, and the MYB–bHLH–WD40 complex, play a central role in regulating anthocyanin biosynthesis by binding to the promoters of structural genes and controlling their transcription (Jaakola *et al.* 2002; Zoratti *et al.* 2014; Dong *et al.* 2014). However, little is known about post-transcriptional regulation of anthocyanin biosynthesis in *C. sinensis*, especially splicing events in the transcriptome.

Alternative splicing (AS), the process of splicing the exons of primary transcripts (pre-mRNAs) from genes in different arrangements, can produce structurally and functionally distinct mRNA and protein variants (Blencowe 2006). Some important phenotype-related genes undergo AS in plants, *e.g.*, those responding to drought, heat (Liu *et al.* 2018c), light (Cheng and Tu 2018), circadian rhythm regulation (Filichkin and Mockler 2012) and flowering time regulation (Macknight *et al.* 2002). Genes in the flavonoid biosynthesis pathway, including *CHS*, *F3H*, *DFR*, *ANS* and *UDP-glucosyltransferase*, also exhibit AS events during kiwifruit (*Actinidia chinensis* ‘Hongyang’) fruit development, whose main pigments in the fruit inner pericarp are anthocyanins (Tang *et al.* 2016). AS of the *ANS* gene, implicated in anthocyanin accumulation, was also detected in variegated peach flowers (Hassani *et al.* 2015). Additionally, AS of *CHS* and *LAR* exists in *C. sinensis* ‘Shuchazao’ (Zhu *et al.* 2018a). We therefore hypothesized that biosynthetic genes may undergo AS, allowing post-translational regulation of anthocyanin biosynthesis in *C. sinensis*.

The PacBio Iso-Seq (isoform sequencing) platform generates long reads—often up to 10 kb—making it possible to accurately reconstruct full-length splice variants. PacBio full-length cDNA sequencing has proven to be a robust technique for discovering thousands of alternatively spliced isoforms in several species, including sorghum, maize, red clover, wheat and rice (Wang *et al.* 2018; Chao *et al.* 2018; Zhang *et al.* 2019). The most extensive efforts to unravel secondary metabolism have been undertaken in *C. sinensis* var. *sinensis* using the single-molecule real-time (SMRT) sequencing platform (Xu *et al.* 2017; Wei *et al.* 2018). In our study, we generated the first full-length transcriptome reference sequences from the ZJ and YK cultivars of *C. sinensis* var. *assamica* derived from purple and green tender shoots, respectively, using the PacBio Iso-Seq technique. In addition, qRT-PCR verification identified more AS isoforms, contributing to our understanding of post-translational regulation of anthocyanin biosynthesis in tea plants.

## Materials and Methods

### Plant materials and RNA sample preparation

Purple young leaves (one bud with two leaves) and green mature leaves of ‘Zijuan’ (ZJ) and young leaves (one bud with two leaves) of ‘Yunkan-10#’ (YK) [both cultivars of *Camellia sinensis* var. *asssamica* (Masters) Kitamura] were sampled, with six replications, from healthy plants grown in the tea garden of the Tea Seed Multiplication Farm of Pu’er City, Yunnan Province, China. All samples were collected in the morning in early August, quickly frozen in liquid nitrogen, and stored at *−*80° until total RNA extraction.

Total RNA was extracted using an Omega Plant RNA kit (Omega Bio-Tek, Code: R6827) according to the manufacturer’s protocol. The quality, integrity and quantity of total RNA were assessed using a NanoDrop 1000 Spectrophotometer (ThermoFisher Scientific, Wilmington, DE, USA) and an Agilent Bioanalyser 2100 with the Agilent RNA 6000 Nano kit (Agilent Technologies, Santa Clara, CA, USA), respectively.

### PacBio Iso-Seq long-read sequencing

First-strand cDNA was synthesized using a Clontech SMARTer PCR cDNA Synthesis Kit (Clontech, Code: 634926), and second-strand cDNA was synthesized by PCR using Phusion High-Fidelity DNA Polymerase (New England Biolabs, Code: M0530). After amplification, PCR products were purified using 0.59 AMPure beads (Beckman, Code: A63880). The products were size-selected using a BluePippin Size Selection System with the following bins for each sample: 1–2, 2–3 and 3–10 kb. Amplified cDNA products were used to generate SMRTbell template libraries in accordance with the Iso-Seq protocol (PacBio). Libraries were prepared for sequencing by annealing a sequencing primer and adding polymerase to the primer-annealed template. Polymerase-bound template was bound to MagBeads, and sequencing was performed on a PacBio RSII instrument by Gene Denovo Biotechnology Co. (Guangzhou, China).

### PacBio data analysis

The SMRT-Analysis software package SMRT Link v5.0.1 (PacBio) was used for Iso-Seq data analysis. Initially, the PacBio raw reads were classified into circular consensus sequence (CCS) and non-CCS sub-reads using ToFu23. The Classify module with default parameters was used to remove adapter sequences, poly-A tails, artificial concatemers and 3′-truncated transcript sequences, resulting in a set of non-full-length isoforms, full-length non-chimeric (FLNC) isoforms, full-length chimeric isoforms and short reads (which were filtered out). For additional error correction, the FLNC transcripts and non-full-length isoforms were analyzed using PacBio Quiver software and Error Correction (ICE). The resulting high-quality (HQ) isoforms were then mapped to the Galgal 4 reference genome assembly using the Genome Mapping and Alignment Program with default parameters.

### Functional annotation of transcripts

Transcripts were annotated by conducting BLASTx searches against public databases, including the NCBI non-redundant protein (Nr), NCBI non-redundant nucleotide (Nt), Swiss-Prot, Protein Family (Pfam), Gene Ontology (GO) and Kyoto Encyclopedia of Genes and Genomes (KEGG) (https://www.kegg.jp/), with an E-value threshold of 10^−5^.

### Identification and classification of AS events

AS events were identified using SUPPA (Alamancos *et al.* 2015), with the {SE,SS,MX,RI,FL} setting for generateEvents and default setting for Boundary. The tool AS talavista (Foissac and Sammeth 2007) was used to classify AS events, resulting in four major AS categories, IR (AS code: 1^2−, 0), ES (AS code: 1–2^, 0), AA (AS code:1−, 2−) and AD (AS code: 1^, 2^), which were identified from the output files and counted. All AS transcripts were used for KEGG enrichment analysis (https://www.kegg.jp/).

### Quantitative RT-PCR

To further analyze the regulatory mechanisms of catechin and anthocyanin biosynthesis, quantitative RT-PCR (qRT-PCR) was performed on the following genes and their AS transcripts: *C4H* (*C4H1*, *C4H2*), *CHS* (*CHS1*, *CHS2*, *CHS3*), *FLS* (*FLS1*, *FLS2*, *FLS3*), *CCOM* (*CCOM1*, *CCOM2*, *CCOM3*), *F3′5’H* (*F3′5’^1^H*, *F3′5’H2*), *LAR* (*LAR1*, *LAR2*), *PAL* (*PAL1*, *PAL2*), *CCR* (*CCR*, *CCR2*), *CYP73A13*, *UDP75L12* (*UDP75L121*, *UDP75L122*, *UDP75L123*), *UDP78A15/UFGT*, *UDP94P1* (*UDP94P11*, *UDP94P12*), *GL3* (*GL3-1*, *GL3-2*), *MYB113* (*MYB113-1*, *MYB113-2*), *ANR*, *ANS*, *F3H*, *4CL1*, *CYP98A3/C3H*, *CHI*, *DFR*. One leaf and two buds and mature leaves from cultivar ZJ and one leaf and two buds from cultivar YK were collected to determine tissue-specific expression levels. qRT-PCR was performed in triplicate according to the 2× SG Green qPCR Mix (with ROX) protocol (SinoGene, Code: Q1002) on a StepOnePLUS Real-Time PCR System (Applied Biosystems, Thermo Fisher Scientific, Waltham, MA, USA) using a 40-cycle program (95° for 20 s and 60° for 30 s). qRT-PCR data were analyzed using the 2^−ΔΔCT^ method (Livak and Schmittgen 2001). All primers used in the present study are listed in Supplementary Table S1.

### Analysis of catechin and anthocyanin content

To analyze the content of catechins and anthocyanins in shoots and leaves, fresh materials were ground to a fine powder in liquid nitrogen. The powder (0.2 g) was extracted with 5 mL methanol at room temperature for 1 h. The residue was re-extracted three times using this method. Supernatants were filtered through a 0.2-μm membrane. Catechins and anthocyanins were analyzed by high-performance liquid chromatography (HPLC) (Wang *et al.* 2012) and quantified at 345 nm and 530 nm, respectively. Statistical analyses based on the data were performed using ANOVA to determine significant differences among group means; data are presented as means ± SD (SD). A *p* value less than 0.05 was considered statistically significant. Correlation analysis between content of catechins and anthocyanins and qRT-PCR was performed using SPSS 22.0. Results are presented using TBtools (http://www.tbtools.com/).

### Data availability

PacBio SMRT sequencing data have been submitted to the SRA of NCBI under accession numbers SRR8639545 and SRR8639546. HPLC chromatography for determation of anthocyanins and catechins (Figure S1 and Table S2, Supplemental data 1). Length distribution of ZJ and YK Iso-seq sub-reads (Supplemental data 2 and Supplementary Figure S2). Number of ZJ (Purple digital) and YK (Green digital) isoforms enriched in terms of molecular function, cellular component and biological process based on GeneOntology (GO) analysis (Figure S3). Gene annotation of KEGG pathways involved in anthocyanin and catechin biosynthesis in ZJ and YK. Isoforms from ZJ and YK were gathered in the KEGG database and their numbers compared (Figure S4). Relative expression quantity of genes and their AS transcripts in ZJ and YK samples with one leaf and two buds compared to ZJ mature leaves based on qRT-PCR studies (Figure S5); primers used are listed (Table S1). Number of isoforms in anthocyanidin biosynthesis of YK and ZJ (Supplemental data 3 and 4). GeneOntology (GO) annotation of ZJ and YK PacBio Iso-seq isoforms (Supplemental data 5) and KEGG annotation of ZJ and YK PacBio Iso-seq isoforms (Supplemental data 6). Classification of AS events in ZJ and YK (Supplemental data 7). Number of AS evens in anthocyanidin biosynthesis in YK and ZJ (Supplemental data 8). Relative expression quantity of genes and their AS transcripts in ZJ and YK samples with one leaf and two buds compared to ZJ mature leaves (Supplemental data 9). Coefficient of association matrix between chemical composition contents and gene expression (Supplemental data 10). Supplemental material available at figshare: https://doi.org/10.25387/g3.10559621.

## Results and Discussion

### Anthocyanin and catechin contents in ZJ and YK

**Figure 1:**
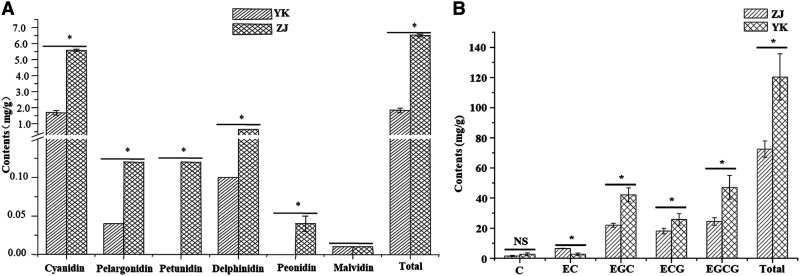
Comparison of contents of anthocyanins and catechins of ZJ and YK. (A) Comparison of contents of anthocyanins of ZJ and YK (B) Comparison of contents of catechins of ZJ and YK; ANOVA results showed the significant (*P* < 0.05) difference and marked marked ‘*’.

Catechin and anthocyanin contents are highly variable in tea plant cultivars (Wang *et al.* 2012). Usually, the content of (−)-Epigallocatechin gallate (EGCG) catechins is the largest, followed by (−)-Epigallocatechin (EGC), (−)-Epicatechin gallate (ECG) and (−)-Epicatechin (EC) in green tea, with (+)-Catechin (C) and (+)-Gallocatechin (GC) catechins usually present in trace amounts (Lachman *et al.* 2003). We detected six anthocyanins (cyanidin, pelargonidin, petunidin, delphinidin, malvidin and peonidin) and five catechins [(+)-C, (−)-(EC), (−)-ECG, (−)-EGC and (−)-EGCG] in leaves of ZJ and YK (Supplementary Figure S1 and Supplementary Table S2). Except for malvidin, the contents of anthocyanins (cyanidin, pelargonidin, petunidin, delphinidin and peonidin) in purple leaves of ZJ were significantly (*P* < 0.05) greater than those in green leaves of YK. Particularly, the total anthocyanin content in ZJ (6.52 ± 0.08 mg/g) was 3.54 times greater than that in YK (1.84 ± 0.14 mg/g) (*P* < 0.05) (Figure 1A). Over 600 anthocyanins have been identified in nature (Smeriglio *et al.* 2016), but just eight main types are found in tea plants (Saito *et al.* 2011). Cyanidin was the main anthocyanidin in both cultivars studied here, and there was no obvious difference in malvidin contents between ZJ and YK. The concentrations of (+)-C, (−)-(EGC), (−)-ECG and (−)-EGCG were greater in YK compared with those in ZJ; however, significantly (*P* < 0.05) more (−)-(EC) accumulated in purple leaves of ZJ (Figure 1B). We identified differential accumulation of anthocyanins and catechins in ZJ and YK, *i.e.*, anthocyanin accumulated more in ZJ, whereas catechins were greater in YK, which is supported by previous work in ‘Mooma 1’ (He *et al.* 2018). Anthocyanins are substrates for the biosynthesis of catechins in plants; the cause of this difference in accumulation of anthocyanins and catechins in purple- and green-leaf cultivars is of great interest.

### Overview of PacBio Iso-Seq of ZJ and YK cultivars

RNA-Seq technologies using the Illumina platform have been extensively used to analyze flavonoid biosynthesis in teas (Wang *et al.* 2018). However, the limitations of short-read sequencing result in the vast majority of isotags not representing full-length cDNA sequences. PacBio Iso-Seq provides reads >10 kb, and up to 60 kb (PacBio, Menlo Park, CA, USA 2016). In order to fully characterize the expression profiles of flavonoid biosynthesis genes, we generated supplemental information using PacBio isoform sequencing of multiple genes. Independent pooled samples of ZJ and YK were sequenced to obtain wide-coverage transcriptomes using PacBio Iso-seq. We obtained 5,150,499 (ZJ) and 5,039,169 (YK) sub-reads based on 7,831,095,131 (ZJ) and 8,013,752,613 (YK) bases sequenced. The length distribution of ZJ and YK Iso-seq sub-reads is displayed in Supplementary Figure S2. We extracted 416,745 (YK) and 405,979 (ZJ) reads of circular consensus sequence (CCS) from the sub-reads. After trimming, assembly, filtering, classifying and clustering, the majority of these [306,188 (73.47%) for YK and 285,067 (70.22%) for ZJ] were full-length reads as indicated by detection of poly (A), as well as 5′ and 3′ primer, sequences. The full-length reads were mapped to the reference genome of the YK cultivar. Full-length non-chimeric sequences (FLNCs) of YK ranged from 131 to 10,547 bp, with an average length of 2,302 bp, whereas FLNCs of ZJ ranged from 130 to 8,823 bp, with an average length of 2,251 bp. The N50 values of the cDNAs were 2,602 and 2,680 bp in ZJ and YK, respectively. Subsequently, 101,178 and 89,438 polished, high-quality (HQ) isoform consensus reads were filtered from 180,132 (YK) and 163,658 (ZJ) full-length cDNAs, respectively, after Quiver polishing using the interactive ICE algorithm.

The isoforms we identified improved the map and annotation, and were similar to those of a previous study (Dong *et al.* 2015). The HQ isoforms obtained for ZJ and YK were mapped to reference genome of ‘Yunkan-10^#^’. Of the total mapped isoforms (87,575/97.92% isoforms for ZJ, 99,884/98.72% isoforms for YK), 13,719/15.34% (ZJ) and 16,620/16.43% (YK) HQ isoforms were multiply mapped, while 73,856/82.58% (ZJ) and 83,264/82.29% (YK) HQ isoforms were mapped to one unique location with greater than 90% coverage and identity, including reads ‘mapped to the positive-sense strand or the negative-sense strand’. The remaining 1863 isoforms from ZJ and 1294 isoforms from YK were unmapped (Table 1). Compared to 80,217 isoforms with an average length of 1,781 bp and N50 of 2,459 bp obtained from *C. sinensis* ‘Shuchazao’, we obtained a higher number of alternative isoforms, which were also longer (Xu *et al.* 2017). The 213,389 polished consensus isoforms have been identified in ‘Yuncha 1’ (*C. sinensis* var. *assamica*) previously (Zhu *et al.* 2018), but the absence of mapping to the reference genome makes these data less reliable than the data we obtained in this paper.

**Table 1:** Overview of results of PacBio Iso-seq

Subjects	Data	Number (%)/length(bp)	
		ZJ	YK
Subreads	Total base (bp)	7831095131	8013752613
	Subreads number	5150499	5039169
	Average length	1520	1590
	N50	2602	2680
Number of CCS	Number of full-length reads	284876	305924
	Full-length non-chimeric, FLNC	278898 (97.90%)	301816 (98.66%)
	Mean full-length non-chimeric read length	2251	2302
HQ isoform mapped to genome	Unique mapped (%)	73856 (82.58%)	83264 (82.29%)
	multiple mapped (%)	13719(15.34%)	16620(16.43%)
	Unmapped (%)	1863 (2.08%)	1294 (1.28%)
Reference transcripts	All mapped Isoforms (%)	23591 (63.84%)	10487 (28.38%)
	Known Isoforms (%)	3293 (13.96%)	3673 (35.02%)
	Novel Isoforms(%)	3819 (16.19%)	2492 (23.76%)
	New Isoforms (%)	16479 (69.85%)	1370 (13.07%)

After annotation using four databases (the non-redundant section of the NCBI database, SwissProt, KEGG and GO databases), 23,591 ZJ isoforms and 28,835 YK isoforms had at least one positive hit in the four public databases. In GO annotation, the majority of isoforms were assigned the molecular functions of protein binding (40% for YK and 41% for ZJ) and catalytic activity (49% for YK and 48% for ZJ), the biological process category of developmental and cellular processes, and the cellular component category of cell formation, in particular, cell membrane, organelle and cell component (15%, 22% and 22%, respectively, in both ZJ and YK) (Supplementary Figure S3).

The isoforms were assigned to 128 and 133 KEGG pathways in ZJ and YK, respectively, with the majority concentrated in carbon metabolism and ribosome pathways (Supplementary Figure S4). We annotated 84, 46, 7, 6 and 1 isoforms in ZJ, and 133, 37, 2, 4 and 1 isoforms in YK into KEGG pathways: Phenylpropanoid biosynthesis (ko00940), Plavonoid biosynthesis (ko00941), Flavone and flavonol biosynthesis (ko00944), Anthocyanin biosynthesis (ko00942) and Isoflavonoid biosynthesis (ko00943), which are directly related to the biosynthesis of flavone, flavonol, catechins and anthocyanins. Full-length cDNAs encoding key genes involved in the biosynthesis of flavonoids were identified, such as *PAL*, *4CL*, C4H, *C3H*, *ANS*, *ANR*, *CHI*, *CHS*, *F3′5’H*, *F3H*, *DFR*, *FLS*, *LAR* and *F3H*. We also identified isoforms involved in modification, stabilization and distribution of anthocyanins (36), for instance, *UDP-glycosyltransferase 78A15*, *5AT*, *3′GT*, *ANL2*, *GL3*, *MYB113* and *AOG* (Table 2). Only 25.68% of full-length cDNAs were known isoforms, with 63.39% newly discovered. In addition, 11.58% were novel isoforms not produced in both ZJ and YK, which may be related to species specificity. A total of 301 isoforms were identified in the flavonoid pathway in *C. sinensis* ‘Shuchazao’ (Xu *et al.* 2017), including more *ANS* (8), *ANR* (8), *F3′H* (7), *DFR* (5), *LAR* (13), but have not the *C3H*, *FLS*, *UDP-glycosyltransferase 78A15*, *5AT*, *3′GT*, *ANL2*, *GL3* and *AOG* isoforms. It should be noted that multiple transcribed sequences for the above genes have also been obtained in *Carthamus tinctorius* L., *Solanum tuberosum* and *Actinidia arguta* (Stushnoff *et al.* 2010; Liu *et al.* 2015; Li *et al.* 2018), which may have been produced by AS events and detected because isoform sequencing provides long reads.

**Table 2:** The annotated isoforms and their AS transcripts of anthocyanidin biosynthesis genes in ZJ and YK

Description	Number of YK Isoforms				Number of ZJ Isoforms				Number of AS transtrips in YK	Number of AS transtrips in ZJ
	new	known	novel	total	new	known	novel	total		
4-coumarate:CoA ligase (4CL1)	5	2	0	**7**	6	3	0	**9**	7	7
aldehyde dehydrogenase (ALDH2C4)	3	0	0	**3**	1	0	0	**1**	3	1
anthocyanidin reductase (ANR)	1	0	1	**2**	1	0	3	**4**	1	1
anthocyanidin synthase (ANS)	0	1	0	**1**	3	1	0	**4**	1	4
Anthocyanin 3′-O-beta-glucosyltransferase (3′GT)	0	1	0	**1**	0	1	0	**1**	2	1
Anthocyanin 5-aromatic acyltransferase (5AT)	0	1	5	**6**	0	0	1	**1**	1	0
Beta glucosidase (BGLU)	16	0	1	**17**	8	0	1	**9**	3	2
Caffeic acid 3-O-methyltransferase (COMT)	0	0	1	**1**	0	0	0	**0**	0	0
Caffeoyl-CoA O-methyltransferase (CCOAOMT1)	5	3	0	**8**	2	2	0	**4**	9	4
caffeoylshikimate esterase (CSE)	15	2	0	**17**	5	2	0	**7**	16	7
chalcone isomerase (CHI)	4	0	1	**5**	5	0	0	**5**	2	1
chalcone synthase (CHS)	2	1	0	**3**	5	1	0	**6**	2	3
cinnamate 4-hydroxylase (CYP73A1)	0	1	0	**1**	1	1	0	**2**	2	2
Cinnamoyl-CoA reductase (CCR)	5	5	0	**10**	6	3	0	**9**	11	5
Cinnamyl alcohol dehydrogenase (CAD)	4	0	0	**4**	8	1	1	**10**	2	4
coumarate 3-hydroxylase (CYP98A3/C3H)	1	0	0	**1**	1	0	0	**1**	1	1
cytochrome P450 81E8-like (CYP81E8)	0	1	0	**1**	1	0	0	**1**	1	1
dihydroflavonol-4-reductase (DFR)	2	0	0	**2**	4	0	1	**5**	2	0
ferulate 5-hydroxylase (CYP84A1/F5H)	0	1	0	**1**	0	0	0	**0**	1	1
flavanone 3-hydroxylase (F3H)	0	1	0	**1**	2	1	1	**4**	2	3
flavonoid 3′,5′-hydroxylase (F3′5’H)	2	0	0	**2**	4	2	0	**6**	2	6
flavonoid 3′-hydroxylase (F3′H)	2	0	0	**2**	0	0	1	**1**	2	1
flavonol synthase (FLS)	2	3	0	**5**	3	2	0	**5**	6	6
AnthocyaninLESS 2 (ANL2)	5	1	0	**6**	5	1	1	**7**	7	7
leucoanthocyanidin reductase (LAR)	1	1	0	**2**	1	1	0	**2**	2	3
myc anthocyanin regulatory protein (GLS)	2	0	0	**2**	4	0	0	**4**	2	4
phenylalanine ammonia-lyase (PAL)	6	5	0	**11**	5	4	0	**9**	9	7
R2R3-MYB transcription factor anthocyanin (MYB113)	1	1	1	**3**	1	0	1	**2**	2	1
shikimate O-hydroxycinnamoyltransferase (HST/SHT)	2	2	2	**6**	1	2	4	**7**	5	4
trans-cinnamate 4-hydroxylase (CYP73A13)	2	0	0	**2**	1	0	0	**1**	1	1
UDP-glycosyltransferase 75L12	0	1	1	**2**	0	2	0	**2**	1	2
UDP-glycosyltransferase 94P1	0	0	2	**2**	0	1	2	**3**	0	0
cytochrome P450 CYP736A54	0	1	0	**0**	0	1	0	**1**	0	0
peroxidase/Cationic peroxidase	28	12	6	**46**	11	8	1	**20**	21	18
UDP-glycosyltransferase 78A15(UFGT)	0	0	0	**0**	0	1	0	**1**	0	1
Total	**116**	**47**	**21**	**183**	**95**	**41**	**18**	**154**	**129**	**109**

### AS transcripts involved in biosynthesis of anthocyanidins

**Figure 2:**
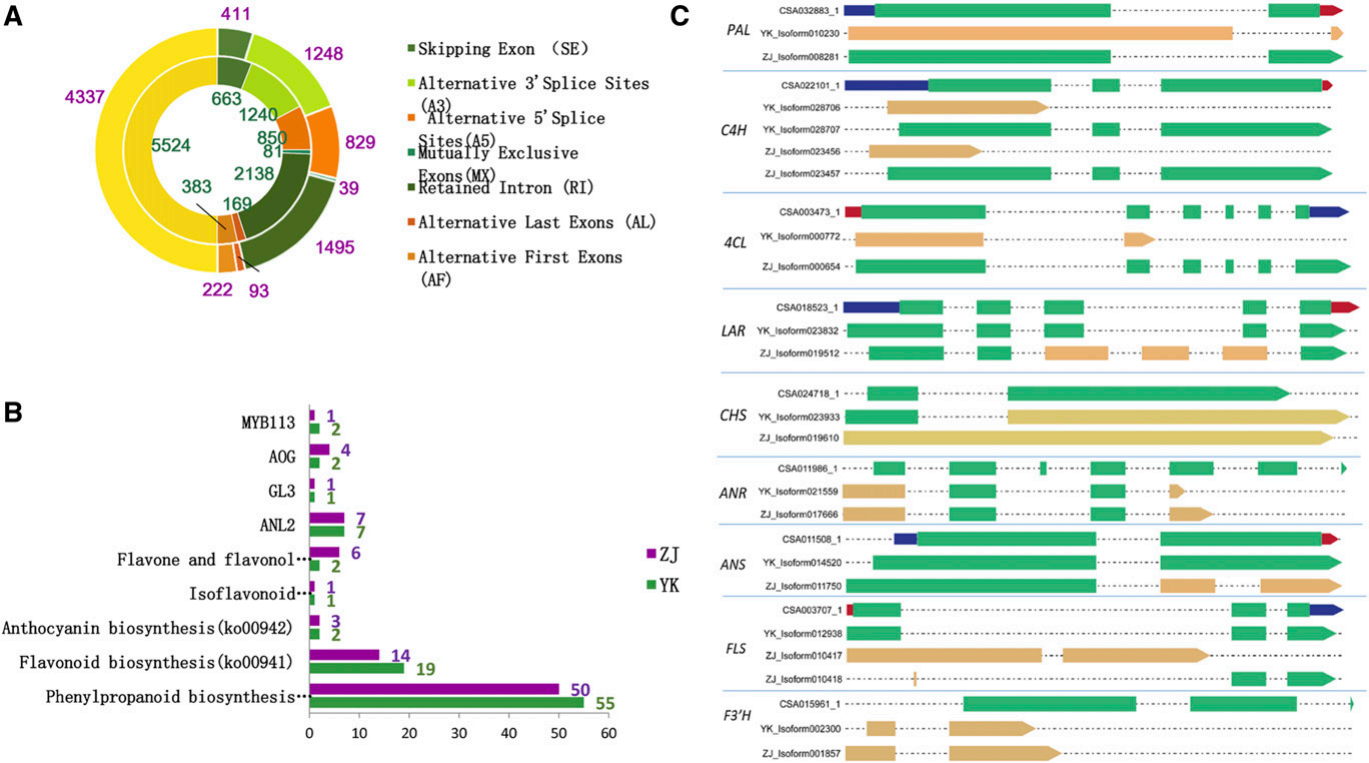
Feature of AS transcripts in *C.sinensis* var *assamica*. (A) Six main types of AS in ZJ and YK, the inner ring with green labeled stand for YK, and outer ring with purple labeled stand for ZJ (B) Number of AS evens screened from PacBio datas annotated into KEGG pathways, as well as *MYB113*, *AOG*, *GL3* and *ANL2* in ZJ (purple font) and YK (green font). (C) AS transcripts of *PAL*, *4CL*, *LAR*, *F3′H*, *CHS*, *ANS*, *ANR*, *LAR*, *FLS* identified using SUPPA.

**Figure 3:**
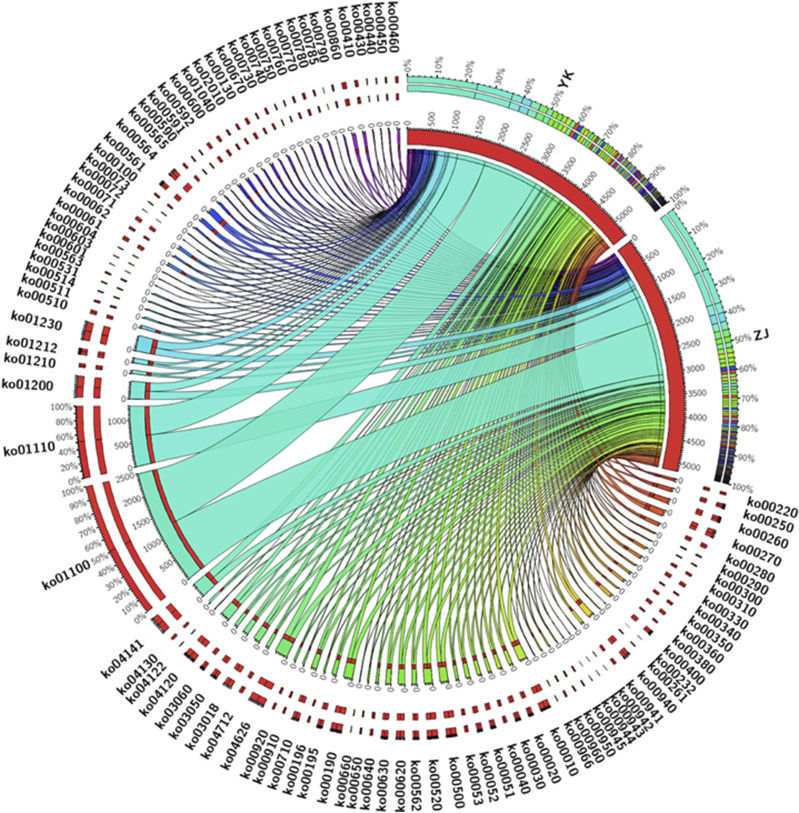
The AS transtripts’ annotation of KEGG pathways involved in anthocyanidin biosynthesis of ZJ and YK.

We detected 4,337 and 5,524 alternatively spliced genes in ZJ and YK from PacBio Iso-seq reads, respectively. Both ZJ and YK generated seven main types of AS: exon skipping, alternative 5′ splice sites, alternative 3′ splice sites, retained introns, mutually exclusive exons, alternative first exon and alternative last exons. Among them, retained introns predominated, accounting for 34.47% and 38.7% of alternative transcripts in ZJ and YK, respectively (Figure 2A, and Supplemental data 7). These alternatively spliced genes were assigned to 131 and 129 KEGG pathways, which were further grouped into 19 classes, including amino acid metabolism, biosynthesis of other secondary metabolites and carbohydrate metabolism. Most alternatively spliced transcripts belonged to the pathway associated with the biosynthesis of secondary metabolites and included genes associated with biosynthesis of amino acids (ko001230), plant-pathogen interaction (ko04626) and purine metabolism (ko00230) (Figure 3). For buds, young leaves, summer mature leaves, winter old leaves, stems, flowers, fruits and roots of *C. sinensis* ‘Shuchazao’, of the 69,569 AS events detected, 20.32% resulted from intron retention (IR), followed by alternative acceptor sites (AA, 15.59%), exon skipping (ES, 15.05%), and alternative donor sites (AD, 14.01%) (Zhu *et al.* 2018a). Thus, tissue-specific AS in tea plants might perform various functions during development. We obtained alternative isoforms that might regulate anthocyanin biosynthesis in tea.

We found that 98 genes annotated with anthocyanin biosynthesis pathways could have undergone AS events (Figure 2B), producing a total of 238 isoforms (Table 2). In YK, 55, 19, two, two and one alternatively spliced genes were associated with the following KEGG pathways related to biosynthesis of anthocyanidins, respectively: phenylpropanoid biosynthesis (ko00940), flavonoid biosynthesis (ko00941), anthocyanin biosynthesis (ko00942), isoflavonoid biosynthesis (ko00943), and flavone and flavonol biosynthesis (ko00944). In ZJ, 50, 14, three, six and one alternatively spliced genes were assigned to the same KEGG pathways mentioned above. These alternatively spliced transcripts encode key enzymes involved in biosynthesis of anthocyanidin, such as PAL, 4CL, LAR, C4H, F3H, CHS, ANS, ANR, LAR, FLS, CCOAOMT, UDP-glycosyltransferase 75L12, beta-glucosidase and peroxidase/cationic peroxidase (Table 2). In addition, alternatively spliced transcripts of *GL3*, *AOG*, *5AT*, *MYB113* and *ANL2* were detected. Interestingly, apart from *DFR* and *5AT* (only detected in YK), AS events occurred in genes of both ZJ and YK, and the alternatively spliced isoforms were different, such as those for transcripts of *PAL*, *4CL*, *F3′H*, *CHS*, *ANS*, *ANR*, *LAR* and *FLS* (Figure. 2C). Therefore, AS events are widespread among biosynthetic genes of anthocyanidin in *C. sinensis* var. *assamica*, suggesting they are involved in the post-transcriptional regulation of anthocyanin biosynthesis as discussed previously (Jia *et al.* 2015). This hypothesis is confirmed by previous reports in other plants showing that AS events make important contributions to gene regulation (Zhu *et al.* 2018) and nearly all structural flavonoid pathway genes generate multiple splice variants (Huang *et al.* 2013).

Homeobox-leucine zipper protein AnthocyaninLESS 2 isoform X2 (*ANL2*), Anthocyanin 3′-O-beta-glucosyltransferase (3′GT) and Anthocyanin 5-aromatic acyltransferase (5AT) are critical modification enzymes. *ANL2* and its mutant variant are homeobox genes from the homeodomain-leucine zipper family that have been proven to reduce accumulation of anthocyanin in subepidermal tissues of *Arabidopsis* (Kubo *et al.* 1999, 2008). We found six and seven *ANL2* gene isoforms in YK and ZJ, respectively, and these may be produced by AS events. 3′GT belongs to the same subfamily as a flavonoid 7-O-glucosyltransferase from *Schutellaria baicalensis* in the plant glucosyltransferase superfamily. It specifically glucosylates the 3′-hydroxy group of delphinidin-type anthocyanins containing Glc groups at the 3 and 5 positions (Fukuchi-Mizutani *et al.* 2003). 5AT catalyzes the transfer of p-coumaric acid and caffeic acid from their CoA esters to the 5-glucosyl moiety of anthocyanidin 3,5-diglucosides, to produce cyanidin 3-glucoside 5-caffeoylglucoside, cyanidin 3-glucoside 5-coumaroylglucoside, delphinidin 3-glucoside 5-caffeoylglucoside and delphinidin 3-glucoside 5-coumaroylglucoside (Fujiwara *et al.* 1997). Higher contents of blue-type anthocyanins were detected in ZJ (0.6545 ± 0.0071 mg/g delphinidin and 5.5793 ± 0.0689 mg/g cyanidin) than in YK (0.0956 ± 0.0023 mg/g delphinidin and 1.6887 ± 0.1402 mg/g cyanidin). Further verification should be carried out to study whether alternatively spliced transcripts of modification enzyme genes (*3′GT* and *5AT*) contribute to this.

### Correlation analysis of anthocyanin biosynthesis and gene expression

After multiple sequence alignment, some genes and their partial alternatively spliced transcripts mentioned above were chosen to test their expression levels using qRT-PCR. The expression profiling shown in Supplementary Figure S5 confirmed that *4CL*, *ANR*, *ANS*, *CYP98A3/C3H*, *C4H*, *CHI*, *DFR*, *F3′5’H*, *FLS*, *LAR*, *PAL*, *CCR*, *CYP73A13*, *UDP75L12*, *UDP78A15*, *UDP94P1*, *CHS*, *CCOM*, *GL3*, *F3H* and *MYB113* exhibited lower expression levels in one leaf and two buds of ZJ than in the same tissues of YK. However, expression of the *F3′5’H*, *F3H*, *FLS*, *LAR*, *CHS*, *GL3* and *MYB113* genes increased in mature leaves of ZJ compared with that in one leaf and two buds. The alternatively spliced transcripts *MYB113-1* and *MYB113-2* showed opposite patterns of expression. In addition, alternatively spliced transcripts of *C4H*, *PAL* and *MYB113* showed discrepant expression in tea plants, with expression of *C4H1*, *FLS1*, *PAL2*, *UDP75L12* and *CCR2* be higher than that of other isoforms.

**Figure 4:**
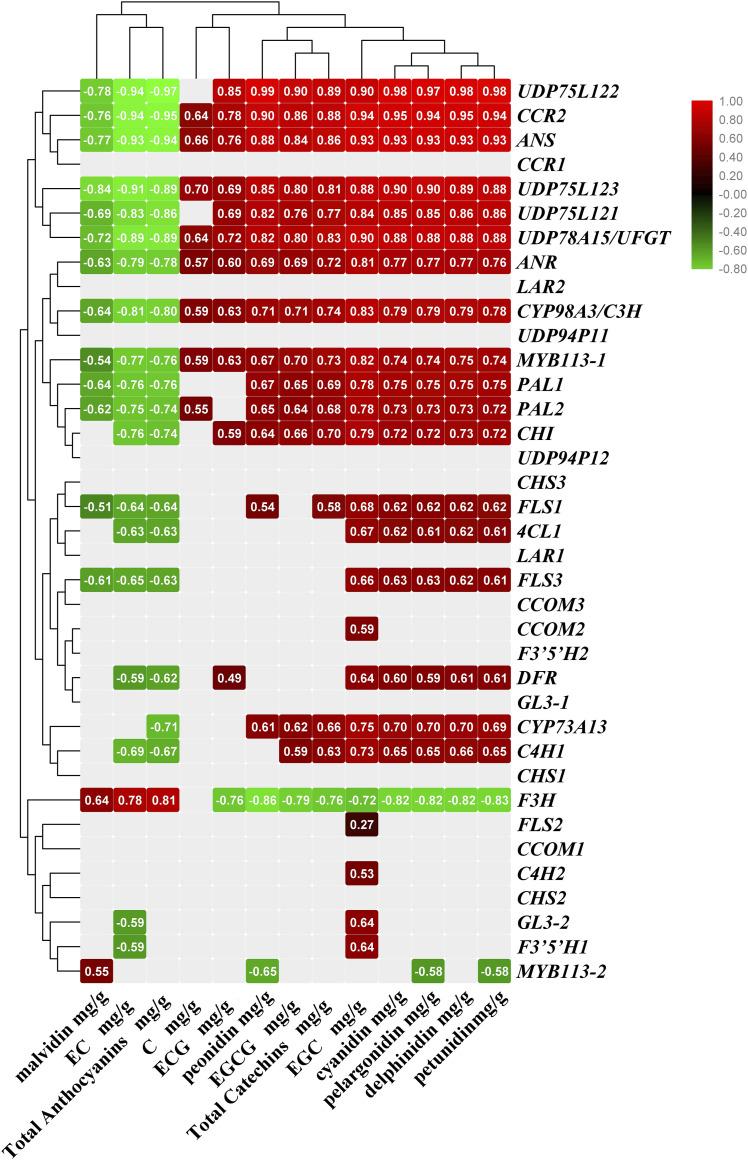
The coefficient of association between chemical composition contents and gene expressions. The number represents the degree of correlation (*P* < 0.05), including highly correlated (0.8-1.0), strong correlated (0.6-0.8), moderate correlated (0.4-0.6), weakly correlated (0.2-0.4), very weakly correlated or uncorrelated (0-0.2). negative number represents negtive correlation and filled with green-series color; positive number represents positive correlation and filled with red-series color, the blank stand for uncorrelated.

To explore the influence of differential expression on anthocyanin content, we analyzed coefficients of association between chemical composition contents and gene expression (Figure 4). Previous studies indicate that alternatively spliced transcripts of *LAR* are significantly correlated (*P* < 0.05) with total catechin content, while those of *PAL* and *ANR* are significantly correlated (*P* < 0.05) with (−)-EGC levels (Zhu *et al.* 2018). However, in this study, catechin and anthocyanin accumulation had nothing to do with *CHS* (*CHS1*, *CHS2* and *CHS2*), *CCOM (CCOM1* and *CCOM3)*, *F3′5’H* (*F3′5’^1^H* and *F3′5’H2*), *LAR* (*LAR1* and *LAR2*), *CCR1*, *UDP94P1* (*UDP94P11* and *UDP94P12*) or *GL3-1*. Total anthocyanin biosynthesis and accumulation showed a significant negative correlation with gene expression (correlation coefficient > −0.8; *P* < 0.05), including that of *C4H1*, *FLS1*, *FLS3*, *4CL*, *ANR*, *ANS*, *CYP98A3/C3H*, *CHI*, *DFR*, *PAL1*, *PAL2*, *CCR2*, *CYP73A13*, *UDP75L12* (*UDP75L121*, *UDP75L122* and *UDP75L123*) and *UDP78A15*, but an extremely significant positive correlation with *F3H* expression (correlation coefficient > 0.8; *P* < 0.01). It is interesting to note that biosynthesis of delphinidin, cyanidin and petunidin showed a significant positive correlation with expression of the genes mentioned above, while malvidin accumulation showed a significant negative correlation with *FLS3*, *ANR*, *ANS*, *CYP98A3/C3H*, *PAL1*, *PAL2*, *CCR2*, *UDP75L12* (*UDP75L121*, *UDP75L122* and *UDP75L123*) and *UDP78A1* gene expression.

Other genes and their alternatively spliced transcripts affected the biosynthesis of catechins, such as *FLS2*, *CCOM2*, *F3′5’^1^H* and *GL3-2*. Expression of most of these transcripts (containing or partly containing *C4H1*, *FLS1*, *FLS3*, *4CL*, *ANR*, *ANS*, *CYP98A3/C3H*, *CHI*, *DFR*, *PAL1*, *PAL2*, *CCR2*, *CYP73A13*, *FLS2*, *CCOM2*, *F3′5’^1^H*, *GL3-2*, *UDP75L121*, *UDP75L122*, *UDP75L123* and *UDP78A15*) showed a positive correlation with EGC and EGCG contents, and a negative correlation with EC contents.

On the basis of the data above, AS isoforms *C4H1*, *FLS1*, *PAL2*, *UDP75L122* and *CCR2* are actively expressed in YK, they show a significant negative correlation with total anthocyanin biosynthesis and accumulation, and show a positive correlation with EGC and EGCG contents; we suggest that these are critical AS transcripts for regulating anthocyanin biosynthesis in *Camellia sinensis var. assamica*.

Most intriguingly, MYB and bHLH TFs were regulated by AS and may serve as activators for mediating downstream genes to affect anthocyanin content (Albert *et al.* 2014; Zhu *et al.* 2018). The expression level of *MYB113* and *GL3* was higher in YK than in ZJ, consistent with the expression pattern described in Arabidopsis (Matsui *et al.* 2008; Zhu *et al.* 2009; Albert *et al.* 2014), so they may downregulate anthocyanin biosynthesis. What is more noteworthy is that AS of *MYB113* TF genes (*MYB113-1* and *MYB113-2)* in this study transcriptionally regulated the chemical composition in opposite patterns: higher expression level of *MYB113-1* in one leaf and two buds of YK than ZJ, but lower expression level of *MYB113-2* in YK. Only *MYB113-1* showed significant positive correlation (*P* < 0.05) with anthocyanin contents in ZJ, and higher expression of *GL3-2* might contribute to more EGC produced in YK than in ZJ.

**Figure 5:**
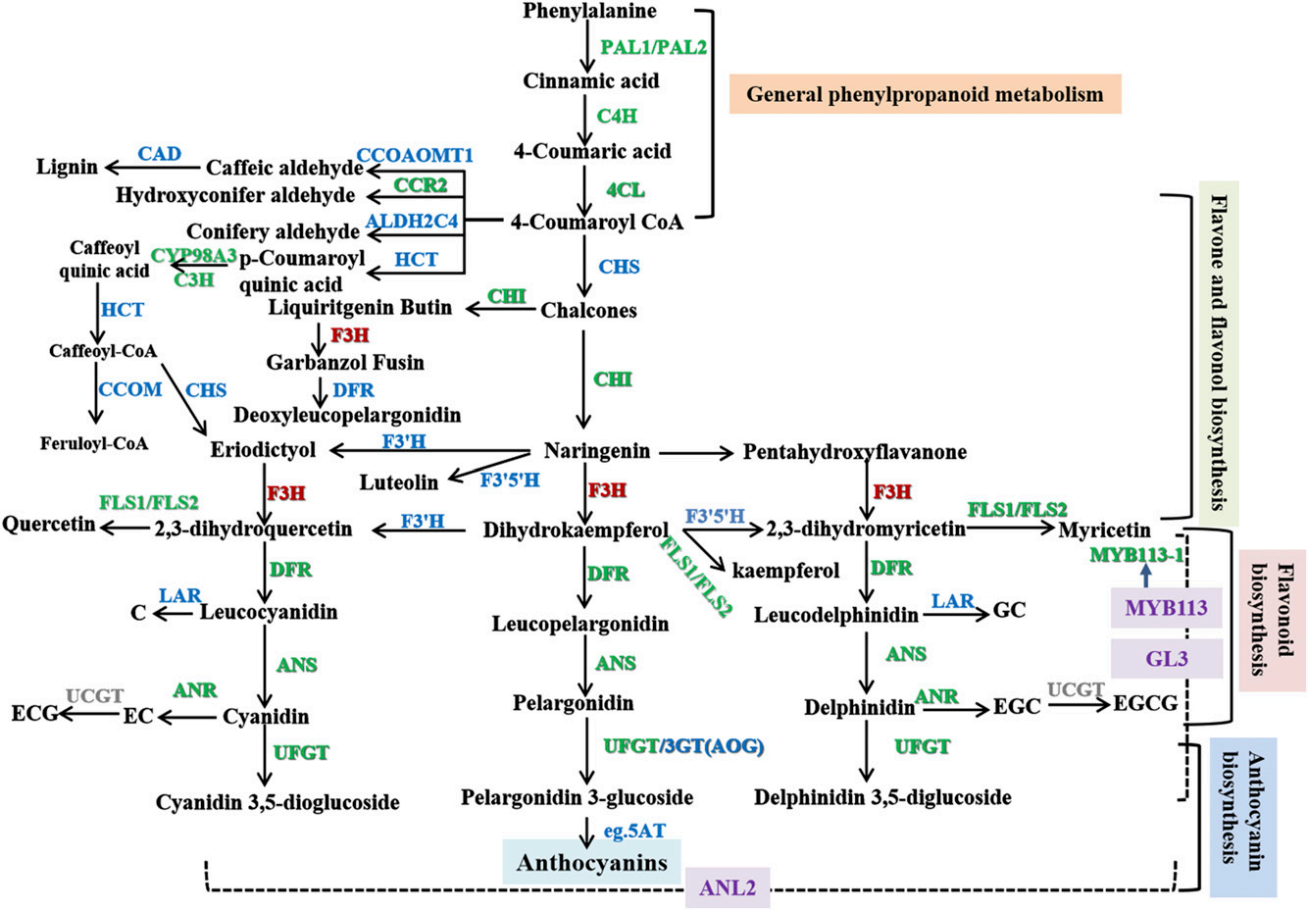
Construction of biosynthesis pathways for anthocyanins in *C. sinensis* var. *Asssamica*. The colorful font represents the genes regulated by alternative splicing evens. the green font represents the genes’ expression are negtive correlation with the total anthocyanins contents, and red font represents the genes’ expression are positive correlation with the total anthocyanins contents.

In summary, we carried out a varietal-specific transcriptome analysis of *C. sinensis* var. *asssamica* using a PacBio long-read sequencing approach to determine the regulatory mechanism of anthocyanin biosynthesis. Alternatively spliced transcripts of genes may act as major mediators at the post-transcriptional level during anthocyanin accumulation in different colorful leaves and cultivars of tea. We speculate here that anthocyanin accumulation in tea plants is most likely due to regulation, *i.e.*, differential expression and AS of transcripts such as *C4H1*, *FLS1*, *FLS3*, *4CL*, *ANR*, *ANS*, *CYP98A3/C3H*, *CHI*, *DFR*, *PAL1*, *PAL2*, *CCR2*, *CYP73A13*, *UDP75L12* (*UDP75L121*, *UDP75L122* and *UDP75L123*), *MYB113-1*, *MYB113-2*, *GL3-2* and *UDP78A15*. *C4H1*, *FLS1*, *PAL2*, *CCR2*, *UDP75L122* and *MYB113-1* are critical AS transcripts for regulating anthocyanin biosynthesis in *Camellia sinensis var. assamica*. The biosynthesis pathways for anthocyanins in *C. sinensis* var. *asssamica* are shown in Figure 5. However, our mechanistic understanding of the roles of AS in tea has remained extremely poor. Continued studies on the functions of regulators will provide new insights into the regulation of anthocyanin metabolism in tea plants.
